# Supplementary material for: Fast saccadic eye-movements in humans suggest that numerosity perception is automatic and direct
Source: Proc Biol Sci. 2020 Sep 23;287(1935):20201884. doi: 10.1098/rspb.2020.1884 (PMC7542817; doi:10.1098/rspb.2020.1884)
Supplement: Supplementary Material to “Fast saccadic eye-movements in humans suggest that numerosity perception is automatic and direct.” [file rspb20201884supp1.docx]

Supplementary Material to “Fast saccadic eye-movements in humans suggest that numerosity perception is automatic and direct.”

Elisa Castaldi^1,2^, David Burr^2,3,*^, Marco Turi^4^ & Paola Binda^1^

^1^ Department of Translational Research and New technologies in Medicine and Surgery, University of Pisa, Pisa, Italy

^2^ Department of Neuroscience, Psychology, Pharmacology and Child health, University of Florence, Italy

^3^ Institute of Neuroscience, National Research Council, Pisa, Italy

^4^ Fondazione Stella Maris Mediterraneo, Potenza, Italy

* corresponding author

Prof. David. C. Burr

**Email:**  davidcharles.burr@unifi.it

**ORCID:** <https://orcid.org/0000-0003-1541-8832>.

Journal name: Proceedings of the Royal Society B

Article DOI: 10.1098/rspb.2020.1884

**Potential confounds: saccadic amplitude**

In the current study participants saccaded to the more numerous of two briefly presented dot arrays. We found that saccadic reaction times were fastest for the intermediate numerical range (estimation range) and slower for the subitizing and density ranges (Figure S1A). Saccadic amplitude can be a potential confound, as faster saccades tend to be shorter than slower saccades^1^. However, the differences in saccadic amplitude cannot explain the difference in saccadic reaction times across regimes: saccadic amplitudes decreased monotonically with numerosity (Figure S1B). This was true when considering all saccades, only the fast and only the slow saccades. Importantly, although we did find, as expected, a general tendency for the fastest saccades to have slightly shorter amplitude, this did not occur for all numerical ranges, as is clear on inspection of Figure S1B. Repeated measures ANOVA on saccadic amplitudes showed a significant interaction between numerical range and fast/slow saccades (F_(2,26)_ = 20.54, p < 0.001) and post-hoc t-tests show that this tendency is most pronounced in the density range (4.9°±0.9° and 5.5°±0.8° for fast and slow saccades; t(13) = 4.7, p=0.003, logBF = 1.9), non-significant in the estimation range (5.2°±0.8° and 5.5°±0.8°; t(13) = 2.4, p=0.4, logBF = 0.3) and absent in the subitizing range (5.6°±0.8° and 5.6°±0.8°; t(13) = 0.3, p=1, logBF = –0.6).

Considering the amplitude of all correct saccades, the effect of numerical range was significant (F_(2,26)_ = 17.65, p < 0.001). However, while average saccadic reaction times vary non-monotonically with numerical range (shortest for the intermediate estimation range), saccadic amplitudes show a monotonically decreasing trend with numerical range (largest for the subitizing range: 5.6°±0.7°, intermediate for the estimation range 5.4°±0.8° and shortest for the density range 5.3°±0.8°).

This indicates that saccadic reaction times and saccadic amplitudes are differentially affected by numerical range. It also shows that fast saccades do not necessarily have smaller amplitudes. Altogether, these results show that differences in saccadic amplitude cannot explain the observed variation of saccadic reaction times across numerical ranges.

**Potential confounds: relative contrast**

RMS contrast (defined as the standard deviation of pixel intensities normalized by the mean) differed between pairs of stimuli across numerical ranges, raising the possibility that the fastest saccades may be driven by stronger contrast differences. We calculated the ratio between all the possible pairs of stimuli for each range. The range of ratios is very similar for the three numerosity conditions. The mean of the ratios increases slightly with numerosity range (Figure S1C), but does so monotonically, while the reaction times are minimal for the estimation range. Thus, it seems unlikely that the observed variation of saccadic reaction times across numerical ranges results from differences in stimulus contrast.

######################################################################


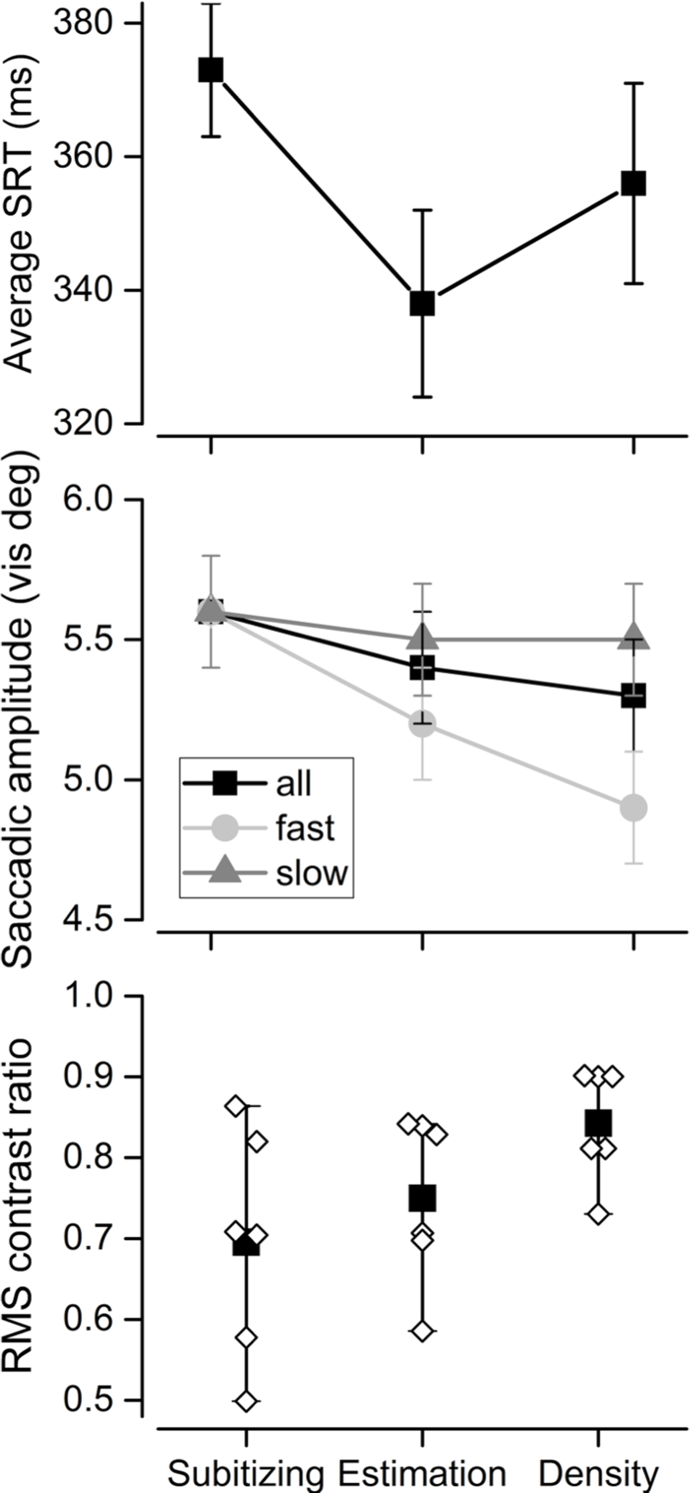


**Supplementary Figure S1: Saccadic reaction times, saccadic amplitudes and stimuli contrast.**

**A.** Average of the reaction-times for correct saccades, as a function of numerosity range. Error bars show ±1 s.e.m. **B.** Average amplitude for the fast, slow and all correct saccades, as a function of the numerosity range. Error bars show ±1 s.e.m. **C.** RMS contrast ratios for all combinations of numerosities within each numerical range (open diamonds). Black squares show the mean ratio, error bars the total range of RMS contrast ratios.

######################################################################

**Distance effects**

Magnitude judgments usually show distance effects: more accurate and faster responses as the numerical distance between stimuli increases. We found evidence for distance effects in all our measures.

Supplementary Figures S2 and S3 plot accuracy and reaction-times of all saccades, of the fastest saccades and of vocal responses, as a function of numerical distance for each numerical range. In all cases accuracy significantly increased with increasing numerical distance between arrays (all saccades: subitizing: F_(2,26)_ = 38.79, p < 0.001; estimation: F_(2,26)_ = 40.54, p < 0.001; density: F_(2,26)_ = 58.40, p < 0.001; fastest saccades: subitizing: F_(2,26)_ = 10.65, p < 0.001; estimation: F_(2,26)_ = 15.36 p < 0.001; density F_(2,22)_ = 49.03, p < 0.001 ; vocal responses: subitizing: F_(2,20)_ = 5.47, p = 0.013; estimation: F_(2,20)_ = 70.47, p < 0.001; density F_(2,20)_ = 37.20, p < 0.001).

Reaction-times also significantly decreased with increasing numerical distance between arrays, when analyzing all saccades (with the exception of the density range which was only close to significance) and vocal responses (all saccades: subitizing: F_(2,26)_ = 14.40, p < 0.001; estimation: F_(2,26)_ = 19.59, p < 0.001; density: F_(2,26)_ = 3.17, p = 0.059; vocal responses: subitizing: F_(2,20)_ = 22.56, p < 0.001; estimation: F_(2,20)_ = 60.75, p < 0.001; density: F_(2,20)_ = 30.35, p < 0.001). In the fastest saccades, the distance effect was significant only in the estimation range (fastest saccades: subitizing: F_(2,26)_ = 1.97, p=0.15; estimation: F_(2,26)_ = 5.71, p = 0.009; density: F_(2,22)_ = 0.59, p = 0.56).

######################################################################


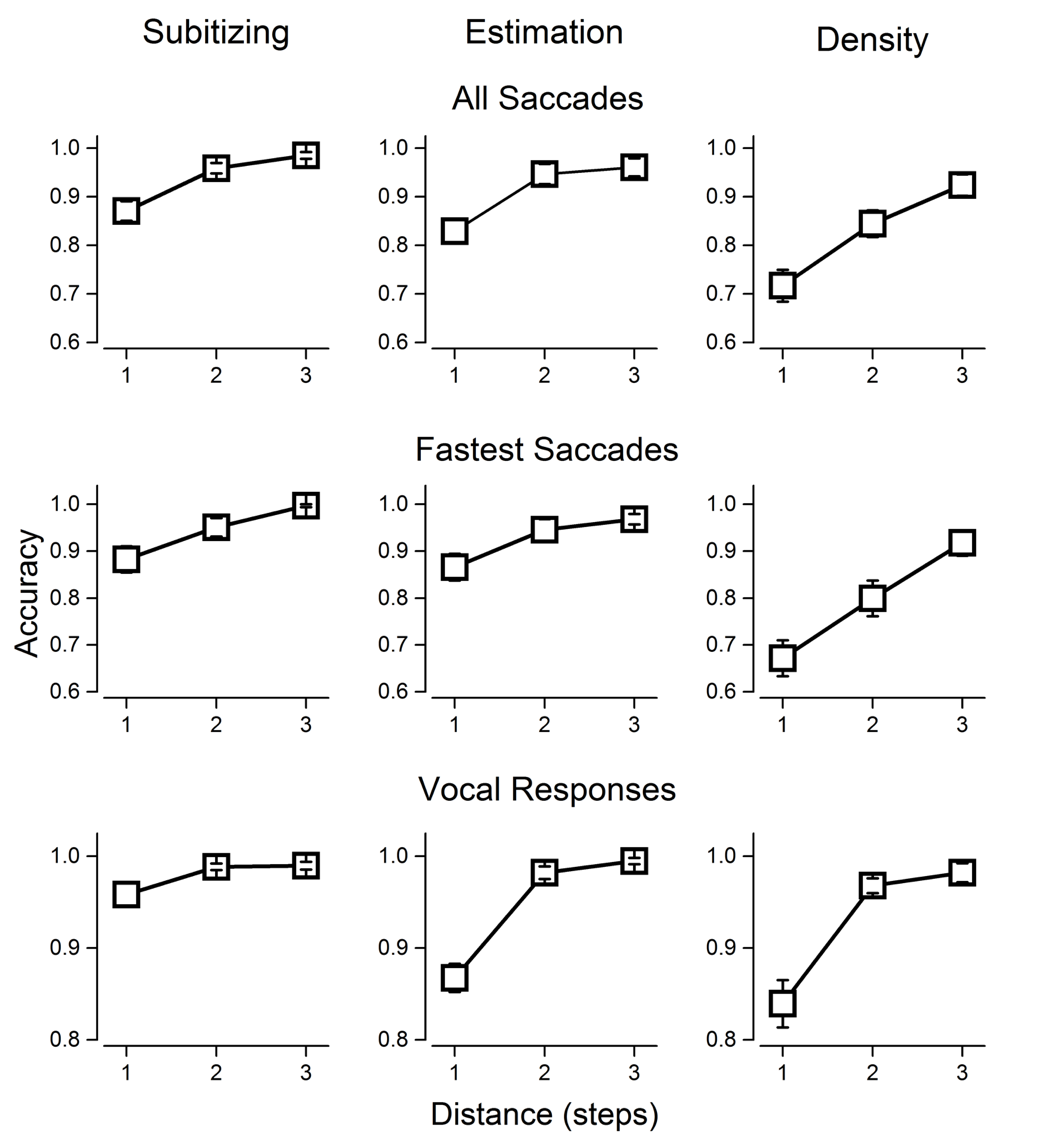


**Supplementary Figure S2: Distance effects.**

Accuracy increased with numerical distance for all saccades (A), fastest saccades (B) and vocal responses (C). Black squares represent mean ±1 s.e.m. Distance “steps” correspond to the numerical distance between numbers (1 to 4) in the subitizing range, and to multiples of 2 JNDs when testing the estimation and density ranges.


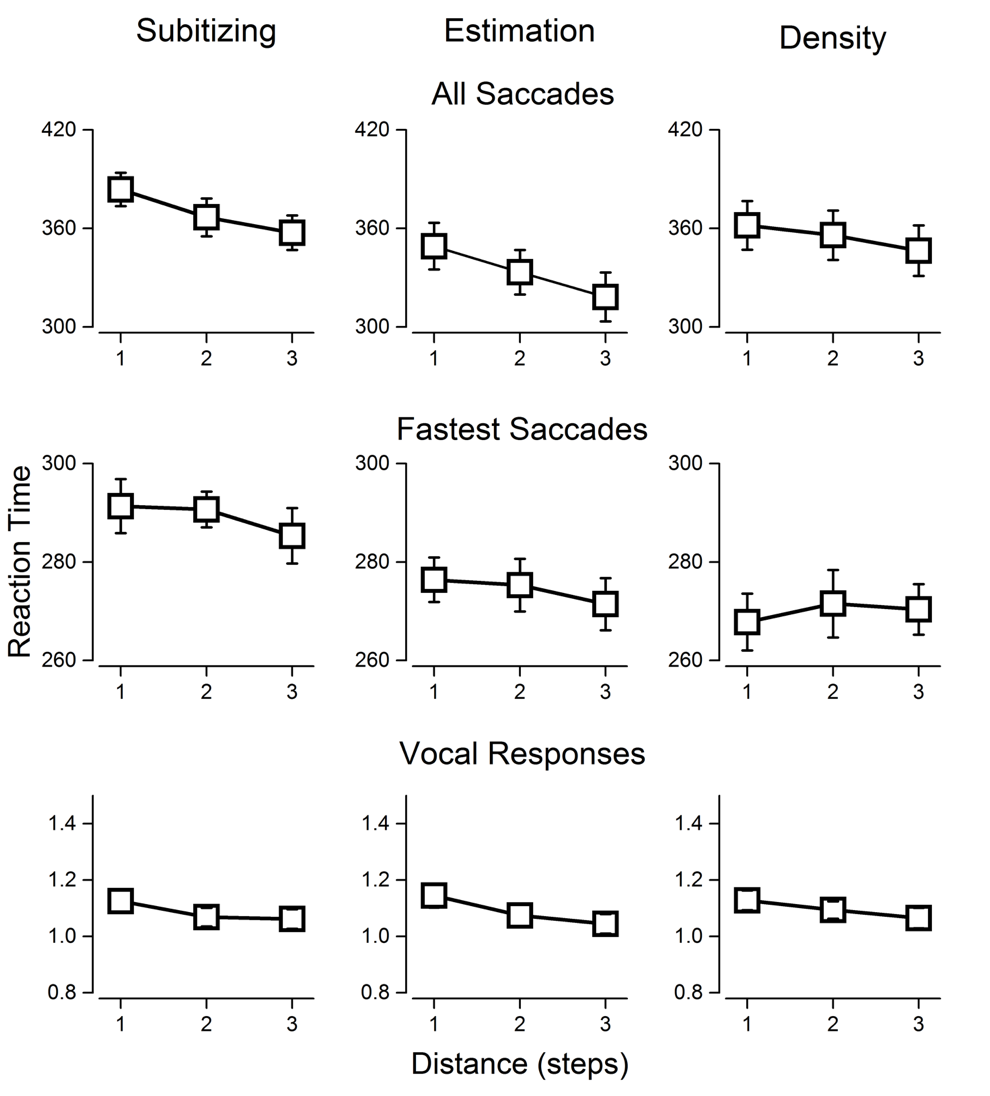


**Supplementary Figure S3: Distance effects – reaction-times**

Reaction-times as a function of numerical distance for all saccades (A), fastest saccades (B) and vocal responses (C). Black squares represent mean ±1 s.e.m.

######################################################################

References:

1. Fuller, JamesH. Eye position and target amplitude effects on human visual saccadic latencies. *Exp Brain Res* **109**, 457–466 (1996).
